# 137. Impact of Antimicrobial Stewardship Interventions on Post-Elective Caesarean Antibiotic Prophylaxis and Surgical Site Infections

**DOI:** 10.1093/ofid/ofab466.339

**Published:** 2021-12-04

**Authors:** Xue Fen Valerie Seah, Yue Ling Rina Ong, Wei Ming Cedric Poh, Shahul Hameed Mohamed Siraj, Kai-Qian Kam, Koh Cheng Thoon

**Affiliations:** 1 KK Women’s and Children’s Hospital, Singapore, Singapore, Singapore; 2 KKH, Singapore, Not Applicable, Singapore; 3 KK Women’s and Children’s Hospital, Singapore, Not Applicable, Singapore

## Abstract

**Background:**

Antimicrobial stewardship programs (ASP) aim to improve appropriate antimicrobial use. Post-operative antibiotics are generally not necessary, especially those without surgical site infections risk factors (e.g. obesity). Few studies have described the impact of ASP interventions on patient outcomes especially in unique populations such as obstetrics. This study aims to evaluate the impact of ASP interventions on post-elective caesarean (eLSCS) oral antibiotic prophylaxis use and patient outcomes including SSI rates.

**Methods:**

This pre-post quasi-experimental study was conducted over 9 months (2 months pre- and 7 months post-intervention) in all women admitted for eLSCS in our institution. Interventions included eLSCS surgical prophylaxis guideline dissemination, where a single antibiotic dose within 60 minutes before skin incision was recommended. Post-eLSCS oral antibiotics was actively discouraged in those without SSI risk factors. This was followed by ASP intervention notes (phase 1) for 3 months, and an additional phone call to the ward team for the next 7 months (phase 2). Phase 3 (next 6 months) constituted speaking to the operating consultant. The primary outcome was post-operative oral antibiotics prescription rates. Secondary outcomes included rates of 30-day post-operative SSI.

**Results:**

A total of 1751 women was reviewed. Appropriateness of pre-operative antibiotic prophylaxis was 98% in our institution. There were 244 women pre-intervention, 274 in post-intervention phase 1, 658 in phase 2 and 575 in phase 3. Pre-intervention post-eLSCS antibiotic prescribing rates was 82% (200), which reduced significantly post-intervention to 54% (148) in phase 1, 50% (331) in phase 2 and 39% (226) in phase 3 (p< 0.001). There was no significant difference in patients who developed post-operative SSI pre-post intervention (0.8%, 2 of 242 vs. 1.9%, 28 of 1479, p=0.420) and among who received post-operative oral antibiotics compared to those without (1.9%, 17 of 905 vs. 1.5%, 13 of 846, p=0.582).

**Conclusion:**

ASP interventions can reduce post-eLSCS antibiotic prophylaxis rates without adversely impacting patient safety.

**Disclosures:**

**All Authors**: No reported disclosures

